# Completing the jigsaw: the first record of the female plant of *Daphnopsisfilipedunculata* (Thymelaeaceae), an endemic species from the Brazilian Amazon

**DOI:** 10.3897/phytokeys.109.28773

**Published:** 2018-10-24

**Authors:** Maurício Takashi Coutinho Watanabe, Nara Furtado de Oliveira Mota, Mayara Pastore, Fernando Marino Gomes dos Santos, Daniela Cristina Zappi

**Affiliations:** 1 Instituto Tecnológico Vale, Rua Boaventura da Silva 955, 66055-090, Belém, PA, Brazil Instituto Tecnológico Vale Belém Brazil; 2 Museu Paraense Emílio Goeldi, Coord. Botânica, Av. Perimetral 1901, 66077-830, Belém, PA, Brazil Museu Paraense Emílio Goeldi Belém Brazil; 3 Amplo Engenharia Gestão de Projetos LTDA, Rua Camões, 28, São Lucas, Belo Horizonte, MG, Brazil Amplo Engenharia Gestão de Projetos LTDA Belo Horizonte Brazil

**Keywords:** Amazon rainforest, Brazil, dioecy, emendation, endemism, Malvales, neotropics, Pará, taxonomy, Thymelaeoideae.

## Abstract

The results of intensive fieldwork in the National Forest of Carajás (FLONA Carajás) led to the discovery of pistillate plants of *Daphnopsisfilipedunculata*, an endemic species from the Serra dos Carajás, previously known only from staminate individuals. These newly discovered populations add valuable missing information related to pistillate buds, mature flowers and fruits.

## Introduction

*Daphnopsis* Mart. comprises 72 species and is the largest American genus of Thymelaeaceae (Rogers 2009−onwards). This Neotropical genus ranges from central Mexico to central Argentina, including the Caribbean (Rogers 2010). The last taxonomic revision of the genus presented an identification key (Nevling 1960); however, as noted by Rogers (2010), both staminate and pistillate flowers were required to key specimens to species. The main difficulty regarding the taxonomy of the genus lies in the fact that its species are dioecious and it often proves difficult to locate specimens of both sexual morphs in the field. More than 20 new species have been described since the last revision almost 60 years ago (Nevling 1960), further complicating identification without complete material.

*Daphnopsisfilipedunculata* Nevling & Barringer is a species that demands special attention. According to its protologue, the systematic position of this species is included in Daphnopsissubg.Neivira, based on its morphological characters, such as axillary inflorescences, monopodial branching and flowers with a lobed disc (Nevling and Barringer 1993). The phylogeny of *Daphnopsis* has not yet been the subject of published molecular studies. Described as restricted to the Brazilian Amazon, *D.filipedunculata* is only known from the Serra dos Carajás (Nevling and Barringer 1993; Mota and Giulietti 2016). In the protologue, the specimens of *D.filipedunculata* were noted to occur in the ecotone between ombrophilous forest and *canga*, a type of vegetation that grows over iron-rich or ferruginous outcrops on the Serra dos Carajás plateau, which contains a mosaic of mostly open vegetation types (Viana et al. 2016). Considered rare, this species was previously recorded only by staminate individuals. According to Mota and Giulietti (2016), the populations appear to have had a large number of plants. However, on closer examination, these “plants” appear to be subterranean sprouts emanating from a single older and more robust individual. Population genetic studies are under way to investigate this hypothesis. The staminate individuals of *D.filipedunculata* are characterised by elliptic leaves and distally developed axillary, subcapitate inflorescences with long peduncles up to 13 cm long (minimum peduncle length measurement ca. 8 cm at anthesis). Staminate flowers have eight stamens attached to the inside of an obconic hypanthium (Nevling and Barringer 1993; Mota and Giulietti 2016).

In this paper, we provide an emended description for *D.filipedunculata* (including vegetative and pistillate data from female individuals), as well as taxonomic notes, photographs and illustrations.

## Methods

Monthly expeditions to the *canga* of the Serra dos Carajás, specifically to the National Forest of Carajás (FLONA Carajás) and surroundings, were carried out over nearly three years, between March 2015 and January 2018, with visits to known localities of *Daphnopsisfilipedunculata* and with targeted searches for additional populations and individuals of the species. The main goal of those visits was to inventory plants for the “Flora of the *canga* of the Serra dos Carajás, Pará, Brazil” (Viana et al. 2016).

Resulting voucher specimens of *Daphnopsisfilipedunculata*, representing staminate and pistillate plants, found in the FLONA Carajás, Pará state, Brazil, were deposited in the herbarium of the Museu Paraense Emílio Goeldi (MG) and Universidade Federal de Minas Gerais (BHCB) (herbarium acronyms according to Thiers 2018). Available duplicates will be distributed to additional national and international herbaria.

For the morphological analysis, all measurements of vegetative parts were taken from dried herbarium collections and field notes. The description of pistillate parts was based on rehydrated specimens dissected under a stereomicroscope (Zeiss Discovery.V8) and measured using a digital pachometer.

The conservation status was evaluated following the IUCN criteria (IUCN 2012; 2017). The parameters extent of occurrence (EOO) and area of occupancy (AOO) were estimated using the GeoCAT software (Bachman et al. 2011) and maps were made using the QGis 2.18 software.

## Results

The protologue description of *Daphnopsisfilipedunculata* was based only on male specimens, making it difficult to associate collections of female individuals with this name. Recently, populations of pistillate individuals were collected in four different areas in the FLONA Carajás (plateaus N1, N2, N3 and N5).

### An emended taxonomic description of *Daphnopsisfilipedunculata*

#### Daphnopsis filipedunculata

Taxon classificationPlantaeMalvalesThymelaeaceae

Nevling & Barringer (1993: 335)

##### Type.

Brazil. Pará: Serra dos Carajás, 6 km SE of AMZA camp N-1, 6°03'S, 50°16'W, 650 m elev., 19 May 1982, *C.R. Sperling et al. 5734* (holotype: F; isotypes: GH, K, MG, MO, NY).

**Figure 1.**
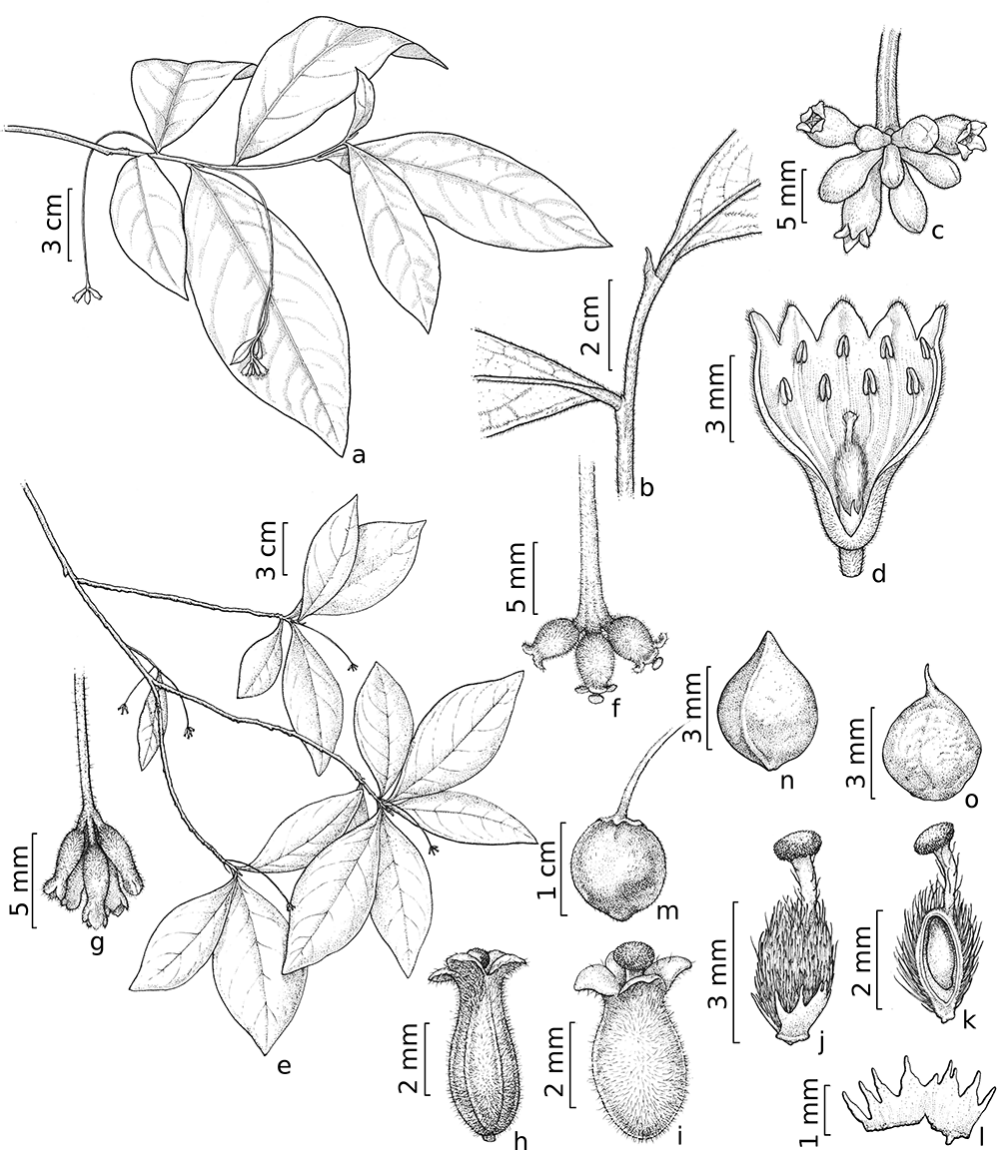
*Daphnopsisfilipedunculata* Nevling & Barringer, male individual (**A–D**) and female individual (**E–O**). **A** staminate flowering branch **B** base of leaf blade **C** detail of inflorescence with staminate flowers **D** dissected staminate flower with two whorls of stamens and one pistillode **E** pistillate flowering branch **F** detail of inflorescence with three pistillate flowers (fresh material) **G** detail of inflorescence with five pistillate flowers (dry material) **H** pistillate flower (dry material) **I** pistillate flower (fresh material) **J** Pistil with disc **K** dissected pistil, longitudinal cut of the ovary showing pendulous ovule **L** details of the lobed disc in female flower **M** fruit **N** seed **O** embryo. Drawn by João Silveira (**A−D** modified from Mota and Giulietti 2016) (**E, G−H, J−L, N−O***Watanabe et al. 485*, **F, I, M***Santos et al. 395* and *396*).

**Figure 2.**
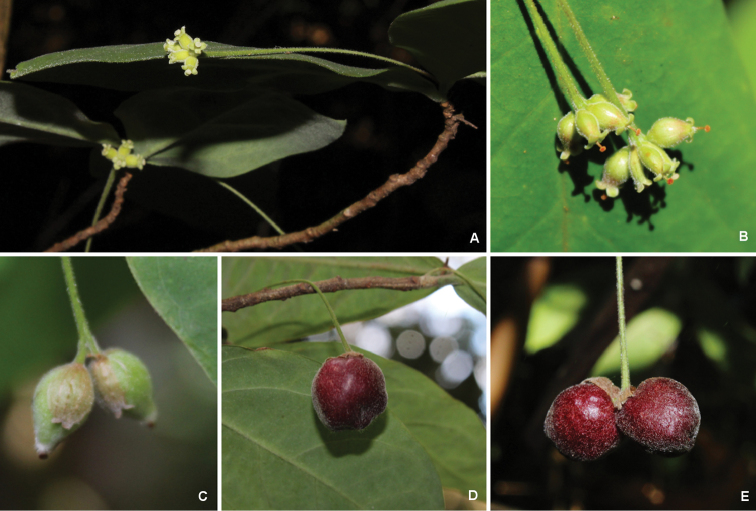
*Daphnopsisfilipedunculata* Nevling & Barringer. **A** pistillate flowering branch **B** detail of inflorescence with pistillate flowers **C** immature fruit with persistent hypanthium **D, E** mature fruits. Photographs by Fernando M. Santos.

Emended description. Pistillate individuals: Shrubs to treelets to 3 m tall. Leaves alternate, concentrated distally on the branches; petioles 1–3 mm long, green to brownish, tomentose, trichomes erect; leaf blade elliptic, 6–13.5 × 2.5–5.5 cm, sericeous on both surfaces, denser on the primary veins; base cuneate; apex acute to acuminate; primary and secondary veins prominent on both surfaces. Pistillate inflorescence axillary, producing densely pilose, subcapitate 3–5-flowered racemes; bracts absent; primary peduncle elongating greatly during anthesis, when flowers opened 3–7.4 cm long, 0.2–0.5 mm wide at the midpoint. Pistillate flowers 3–5 mm long; hypanthium urceolate, 2–4 mm long, 2–3 mm wide at opening/constriction, pubescent outside, trichomes erect; calyx lobes 4, triangular to rounded, ca. 1 mm long, ca. 0.5 mm wide, revolute; staminodes absent; disc free, irregularly 6–8–lobed, the lobes acute, ca. 0.5 mm long; gynoecium 3–4 mm long, style 1–2 mm long, subterminal, stigma discoid, ovary ovoid, unilocular, 1–1.2 mm wide, densely pilose. Fruits berry-like, green to yellow initially, turning dark red when ripe, irregularly globose, 5–15 × 4–12 mm, pilose, trichomes white to greyish, the style and hypanthium often persistent, tearing regularly, longitudinally as fruit matures. Seeds pyriform, 2–3 × 4–5 mm. (Figures 1, 2)

##### Material examined.

Brazil. Pará: Parauapebas, Floresta Nacional de Carajás–Serra dos Carajás, Serra Norte, Plateau N1, mata baixa, 21 June 2012 [staminate fl.], *L.C.V. Silva et al. 1263* (MG); Plateau N1, 6°02'46"S, 50°16'06"W, 693 m elev., 26 May 2017 [staminate fl.], *F.M.G. Santos et al. 392* (MG); Plateau N1, 06°02'46"S, 50°16'06"W, 693 m elev., 31 May 2017 [pistillate fl.], *F.M.G. Santos et al. 393* (MG); Plateau N1, 06°02'45"S, 50°16'06"W, 679 m elev., 30 May 2017 [pistillate fl.], *F.M.G. Santos et al. 394* (MG); Plateau N1, 06°02'46"S, 50°16'06"W, 693 m elev., 31 May 2017 [pistillate fl.], *F.M.G. Santos et al. 396* (MG); Plateau N1, 6°02'43.2"S, 50°14'44.3"W, 8 Aug. 2017 [pistillate fl.], *M.T.C. Watanabe et al. 485* (BHCB, MG, RB); Plateau N2, 06°03'38"S, 50°14'46"W, 707 m elev., 24 Aug. 2012 [staminate fl.], *A.J. Arruda et al. 1269* (BHCB); Plateau N2, 06°03'09"S, 50°15'21"W, 705 m elev., 26 Jul 2017 [staminate fl.], *F.M.G. Santos et al. 397* (MG); Plateau N2, 06°03'09"S, 50°15'21"W, 697 m elev., 26 July 2017 [pistillate fl.], *F.M.G. Santos et al. 398* (MG); Plateau N3, 21-31 July 2017 [staminate fl.], *L.C.B. Lobato 4298* (MG); Plateau N3, 06°02'44"S, 50°12'35"W, 716 m elev., 30 May 2017 [pistillate fl.], *F.M.G. Santos et al. 395* (MG); Plateau N3, 06°02'45"S, 50°12'29"W, 714 m elev., 30 May 2017 [fruit], *F.M.G. Santos et al. 399* (MG); Plateau N3, 06°02'45"S, 50°12'30"W, 714 m elev., 26 June 2017 [pistillate fl.], *F.M.G. Santos et al. 400* (MG); Plateau N3, 06°02'47"S, 50°12'28"W, 720–869 m elev., 26 Jun 2017 [staminate fl.], *F.M.G. Santos et al. 401* (MG); Plateau N3, Floresta ombrófila aberta, 06°02'45"S, 50°12'30"W, 706 m elev., 22 June 2017 [staminate fl.], *M.V. Waldir 276* (MG); Plateau N4, 06°06'54"S, 50°11'03"W, 678 m elev., 01 June 2017 [staminate fl.], *F.M.G. Santos et al. 402* (MG); Plateau N4, 06°06'55"S, 50°11'02"W, 680 m elev., 1 June 2017 [staminate fl.], *F.M.G. Santos et al. 403* (MG); Plateau N5, 06°06'15"S, 50°08'13"W, 706 m elev., 31 May 2017 [staminate fl.], *F.M.G. Santos et al. 404* (MG); Plateau N5, Lagoa da Mata, 06°02'29"S, 50°05'18"W, 664 m elev., 1 June 2017 [staminate fl.], *F.M.G. Santos et al. 408* (MG); Plateau N5, Lagoa da Mata, 06°02'29"S, 50°05'18"W, 664 m elev., 24 March 2017 [staminate fl.], *F.M.G. Santos et al. 409* (MG); trilha para Lagoa, plateau N5, 06°02'28.5"S, 50°5'15"W, 667 m elev., 21 June 2015 [staminate fl.], *R.M. Harley et al. 57246* (MG); Plateau N5, Aug. 2017 [fr], *M.T.C. Watanabe et al. 482* (MG) Plateau N5, trilha da mata, 06°03'20"S, 50°05'15"W, 682 m elev., 14 Aug. 2016 [staminate fl.], *L.V. Vasconcelos & M.E.L. Lima 933* (MG); Plateau N6, 06°07'33"S, 50°10'44"W, 704 m elev., 1 June 2017 [staminate fl.], *F.M.G. Santos et al. 405* (MG); Plateau N7, 06°09'12"S, 50°10'22"W, 677 m elev., 1 June 2017 [staminate fl.], *F.M.G. Santos et al. 406* (MG); Plateau N7, 6°09'13"S, 50°10'20"W, 697 m elev., 1 June 2017 [staminate fl.], *F.M.G. Santos et al. 407* (MG); Serra dos Carajás, 6 km SE of AMZA camp, 6°03'S, 50°16'W, 650 m elev., 19 May 1982 [staminate fl.], *Sperling et al.* 5734 (F, GH, K, MG, MO, NY).

##### Taxonomic notes.

Vegetative parts of pistillate plants of *Daphnopsisfilipedunculata* are very similar to male plants. Staminate flowers have pistillodes and a deeply 4–lobed disc, while the pistillate flowers lack staminodes and the disc is irregularly 6–8–lobed. The female inflorescences bears three to five flowers, however only one or two of these usually develop into fruit (Figures 1, 2). Figure 1A (adapted from Mota and Giulietti 2016) represents a staminate individual with a floral bract and branched peduncle; however, through analysis of the above-listed material, it was found that only a single duplicate of *Harley et al. 57246* (unfortunately the one selected to be drawn) shows these characters. No other specimen had bracts, not even bract scars and none had apically branched peduncles. Therefore, we believe that the bract illustrated in Mota and Giulietti (2016) is an unusual feature uncommonly found in this species.

Populations are formed of isolated clumps of individuals separated by small distances (20–50 m), which makes it difficult to define the limits between adjacent individuals. Within a clump, sometimes individuals are found growing very close together and sometimes found suckering, presenting clonal behaviour that presumably also occurs in other groups of Thymelaeaceae, such as the African and Malagasy *Gnidia* L. (Rogers 2009). It is impossible to distinguish morphologically between individuals of different sexes of *D.filipedunculata* unless they are fertile, but during our studies, we observed that pistillate individuals are normally found at lower frequencies than staminate ones. Fertile specimens were collected between May and August, with occasional fertility in March in both sexes.

##### Distribution and habitat.

Endemic from the *canga* of the Serra dos Carajás, this species is restricted to the formation known as Serra Norte, where it grows in low, deciduous forest over ferruginous soil (Mota and Giulietti 2016) in transitional areas between open *canga* and ombrophilous forest. It is restricted to the plateau areas, having never been found in open forests on the slope. Populations with individuals from both sexes were found on all iron rock outcrops plateaus from N1 to N7 and an additional population was found only to contain male individuals on a small *canga* outcrop area known as Lagoa da Mata.

##### IUCN preliminary conservation assessment.

*Daphnopsisfilipedunculata* has only been recorded within the FLONA Carajás, at the Serra Norte (N1, N2, N3, N4, N5, N6 and N7). According to the criteria set by IUCN (2012, 2017), the species is assigned a preliminary conservation assessment of Endangered [EN B1ab(i,ii,iii)+2ab(i,ii,iii); D], as it has a restricted distribution with an extent of occurrence (EOO) of 159.28 km^2^ and an area of occupancy (AOO) of 44 km^2^, based on a cell size of 4 km^2^. The total population size is estimated as fewer than 250 mature individuals. In addition to being known from a single general site, the species has an inferred and projected decline in both its EOO and AOO and in its quality of habitat that is located adjacent to the mining area within the FLONA Carajás. Conservation proposals are currently being elaborated for this species in the management plan of this sustainable use protected area.

**Figure 3.**
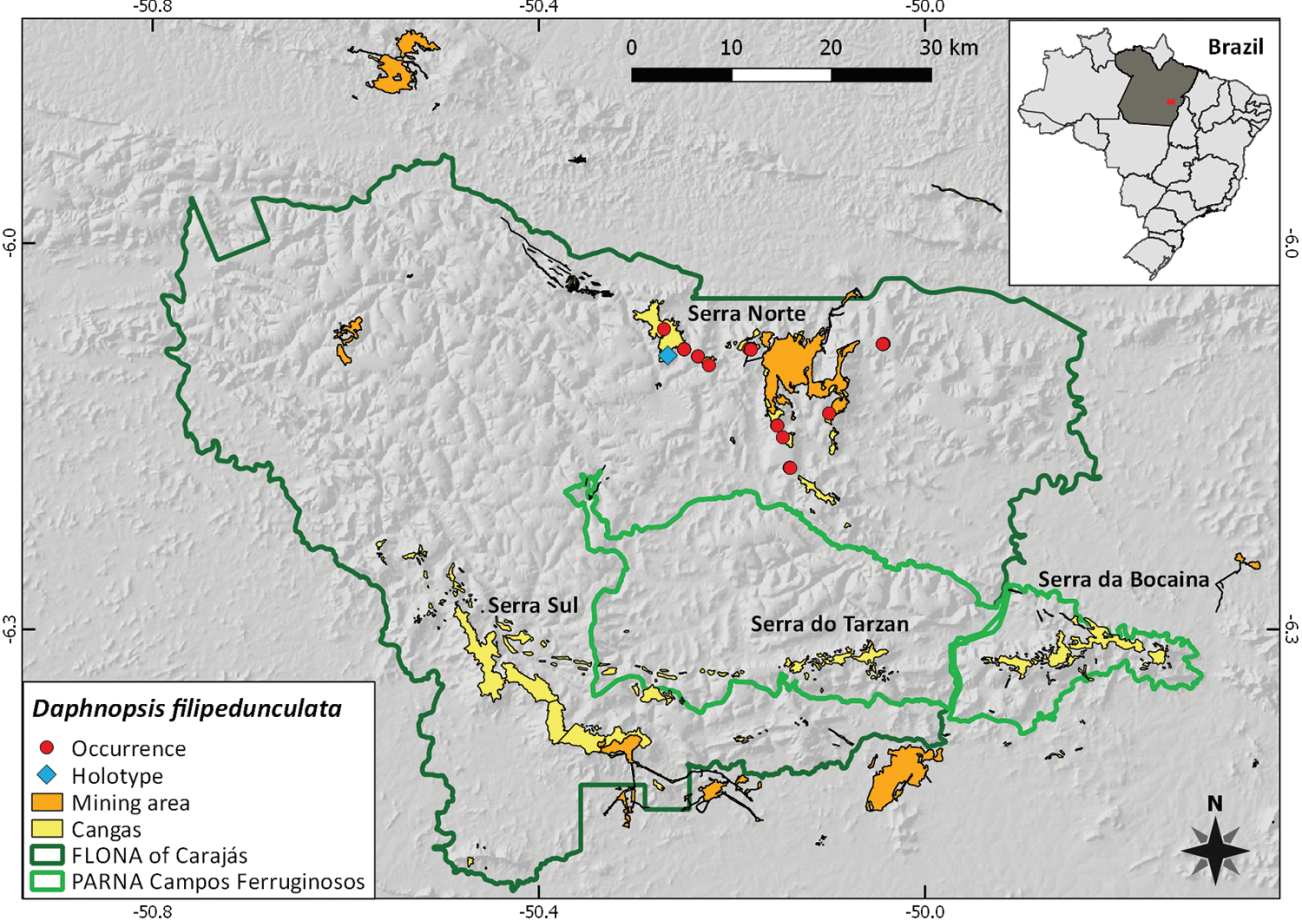
Distribution of *Daphnopsisfilipedunculata* Nevling & Barringer along the Serra dos Carajás.

**Figure 4.**
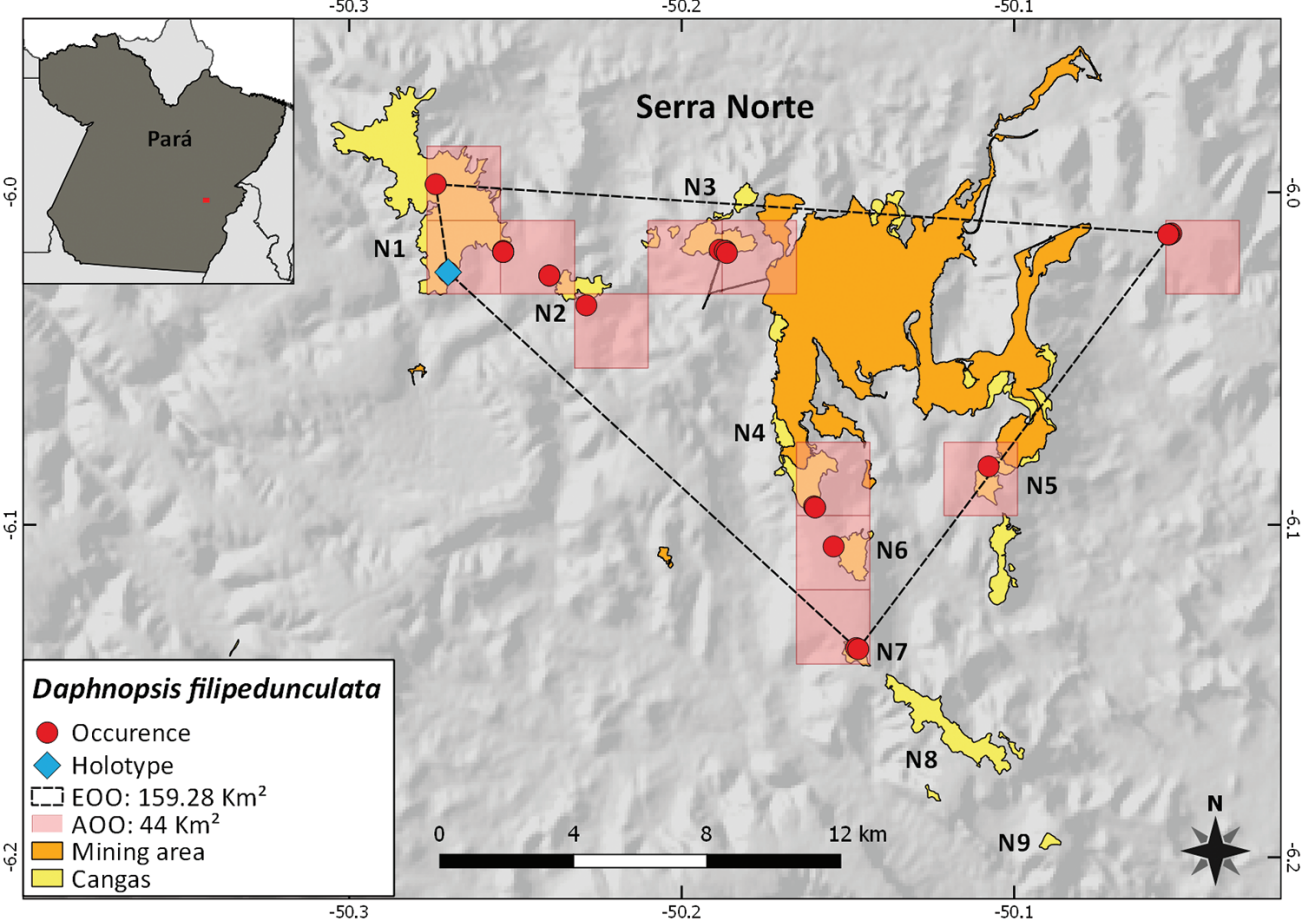
Distribution of *Daphnopsisfilipedunculata* Nevling & Barringer along the Serra dos Carajás with convex polygon and grids of 4 km^2^.

## Supplementary Material

XML Treatment for Daphnopsis filipedunculata
